# Supplementary figures and images for: Genome-Wide DNA Methylation and Gene Expression Profiles in Cows Subjected to Different Stress Level as Assessed by Cortisol in Milk
Source: Genes (Basel). 2020 Jul 25;11(8):850. doi: 10.3390/genes11080850 (PMC7464205; doi:10.3390/genes11080850)

**Figure S2.** Overlap of DMGs and DEGs in the two High and Low cortisol groups.

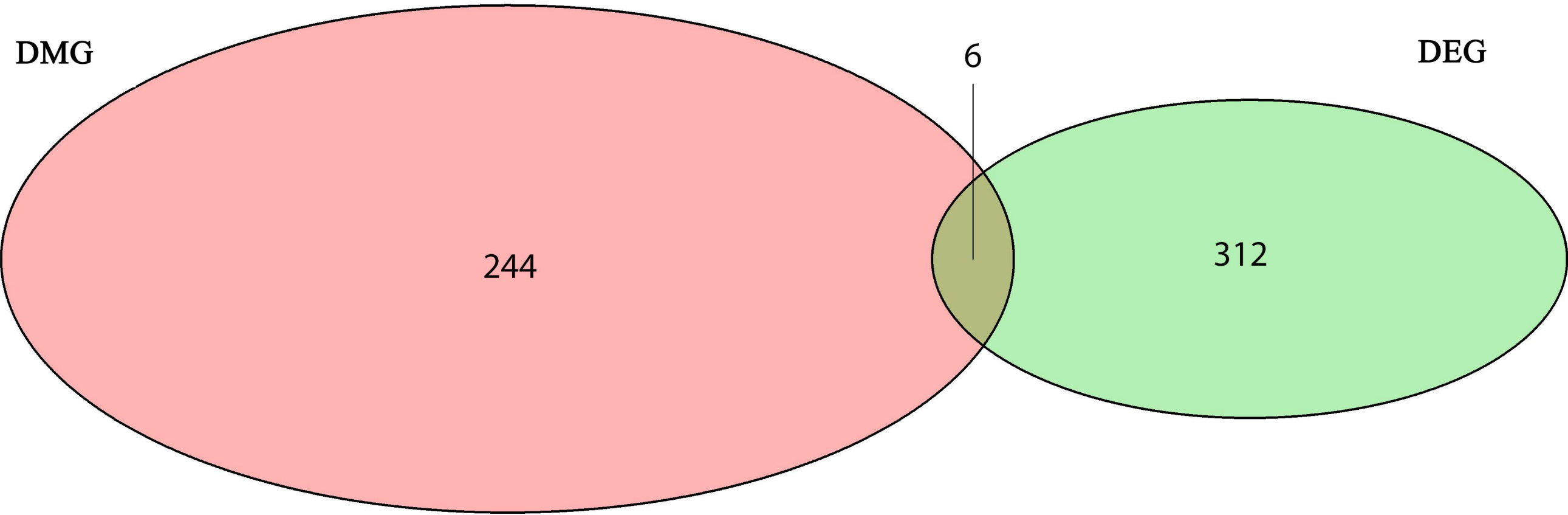

Supplement: Supplementary file 1 [file genes-11-00850-s001.zip › Supplementary Fig. S1.pdf]
